# Use of medicinal plants as a remedy against lymphatic filariasis: Current status and future prospect

**DOI:** 10.1002/hsr2.1295

**Published:** 2023-05-27

**Authors:** Fatima A. Fordjour, Priscilla Osei‐Poku, Afua K. A. Genfi, Kwaw G. Ainooson, Kingsley Amponsah, Patrick K. Arthur, G. Richard Stephenson, Alexander Kwarteng

**Affiliations:** ^1^ Department of Microbiology University for Development Studies Tamale Ghana; ^2^ Department of Biochemistry and Biotechnology, College of Science Kwame Nkrumah University of Science and Technology Kumasi Ghana; ^3^ Kumasi Centre for Collaborative Research in Tropical Medicine Kwame Nkrumah University of Science and Technology Kumasi Ghana; ^4^ Department of Biochemistry University for Development Studies Tamale Ghana; ^5^ Department of Pharmacology, Faculty of Pharmacy and Pharmaceutical Sciences Kwame Nkrumah University of Science and Technology Kumasi Ghana; ^6^ Department of Pharmacognosy, Faculty of Pharmacy and Pharmaceutical Sciences Kwame Nkrumah University of Science and Technology Kumasi Ghana; ^7^ Department of Biochemistry, Cell and Molecular Biology University of Ghana Accra Ghana; ^8^ School of Chemistry University of East Anglia Norwich UK

**Keywords:** antibiotics, anticancer, anti‐inflammatory, lymphatic filariasis, medicinal plants

## Abstract

Despite the successes achieved so far with the Global Programme to Eliminate Lymphatic Filariasis, there is still an appreciable number of lymphatic filarial patients who need alternative treatment and morbidity management strategies. The unresponsiveness of some cohorts to the drugs used in the mass drug administration program is currently raising a lot of questions and this needs urgent attention. Natural medicinal plants have a long‐standing history of being effective against most disease conditions. Countries such as India have been able to integrate their natural plant remedies into the treatment of lymphatic filarial conditions, and the results are overwhelmingly positive. Components of *Azadirachta indica A. Juss, Parkia biglobosa*, *Adansonia digitata*, and *Ocimum* spp have been shown to have anti‐inflammatory, anticancerous, and antimicrobial activities in animal models. Therefore, this review calls for attention toward the use of natural plant components as an alternate treatment against lymphatic filariasis to help reduce the World Health Organization's burden of providing drugs for people in need of treatment every year.

## 1 INTRODUCTION

**Figure 1:**
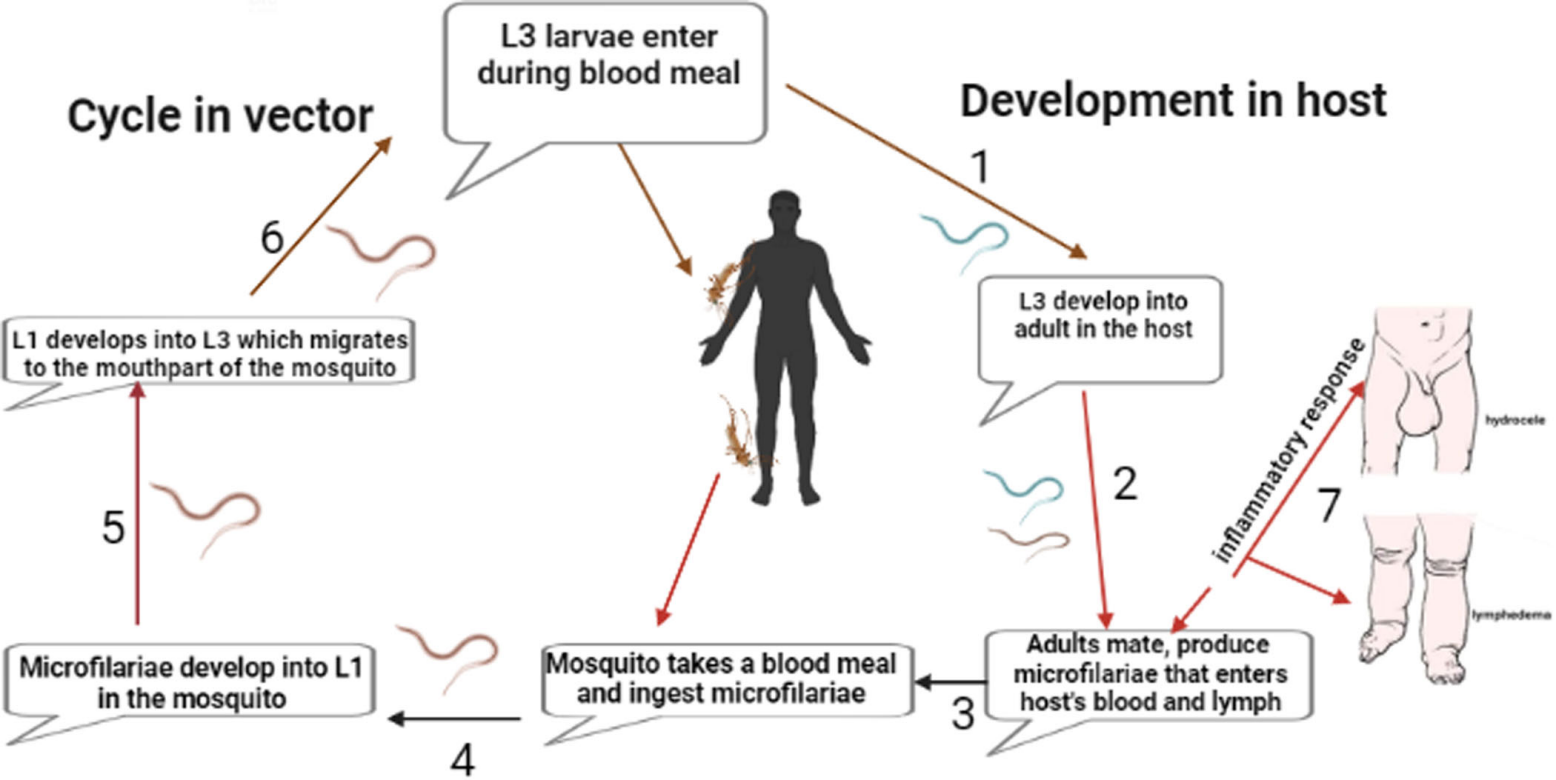
Lifecycle of filarial parasites, demonstrated with *W. bancrofti*. Both a vector and a mammalian host are required for the development of the nematode. (1) The infected vector transmits the third‐stage larvae (L3) into the human host during a blood meal. (2) The L3 mature into adult worms. (3) The parasites produce microfilariae (MF), which migrate to the lymphatics and blood for circulation. (4) The vector once again ingests the microfilariae during a blood meal from an infected host. (5) The microfilariae develop into the L1 stage. (6) The L1 larvae matures into L3 larvae which migrate to the vector's proboscis via the haemocel. (7) Clinical manifestations of LF due to inflammation mainly from worm antigen. Created with Biorender.com.

Lymphatic filariasis (LF) is a neglected tropical disease (NTD) that occurs when filarial parasites (*Wuchereria bancrofti* and *Brugia* spp) are transmitted to humans through mosquitoes (Figure 1). LF is a vector‐borne, long‐standing chronic disease which is the second leading cause of long‐term and permanent disability in the world.^1^ This disease has been a public health concern, especially in Africa and South America. LF is caused by the lymph–dwelling nematode parasites; *W. bancrofti*, *Brugia malayi*, and *Brugia timori*. The filarial nematode *W. bancrofti* accounts for 91% of LF infections while *B. malayi* and *B. timori* are responsible for the remaining 10% in South and Southeast Asia.^2^, ^3^, ^4^ The clinical manifestations of the LF include hydrocele and lymphedema (LE) (mainly known as elephantiasis).

The development of modern medicines for NTDs from phytochemicals is intrinsically less likely to fail through problems of toxicity or other side effects when the phytochemical lead compounds originate from plant species, which are part of certificated herbal treatments.^5^ Many have been monitored over a number of years/decades in clinical use with significant number of patients, for health conditions which are more serious than the NTD against which the phytochemicals could also shows promising activity. The current World Health Organization's (WHO) drugs of choice for its mass drug administration (MDA) program are ivermectin (IVM), albendazole (ALB), and diethylcarbamazine (DEC).^6^ These drugs are effective in reducing microfilariae but not effective in killing the adult worms or relieving individuals with morbidity.^7^, ^8^ Furthermore, there is repopulation of microfilariae in the host system 6–12 months after treatment, hence the need to take the drugs annually. DEC has also been reported to cause severe side effects such as fever, gastrointestinal disturbance, headache, malaise, and skin rash that reduce patient's compliance.^9^ The pathology is known to progress due to elevated inflammatory processes that unfolds in the presence of filarial antigen.^10^, ^11^, ^12^

Data from field trials have shown potency of some antibiotics (doxycycline and minocycline) against LF.^7^, ^13^, ^14^, ^15^, ^16^ This notwithstanding, these antibiotics have a longer regimen duration and do not support national treatment programs. Thus, there is still the need for more effective drugs with less adverse reaction, shorter regimen duration, and less cost to achieve WHO's aim of interrupting transmission and helping individuals with LF morbidity. Moreover, these antibiotics usually clear microfilaria with a delayed gradual effect on the early stages of pathology (hydrocele and LE).

**Figure 2:**
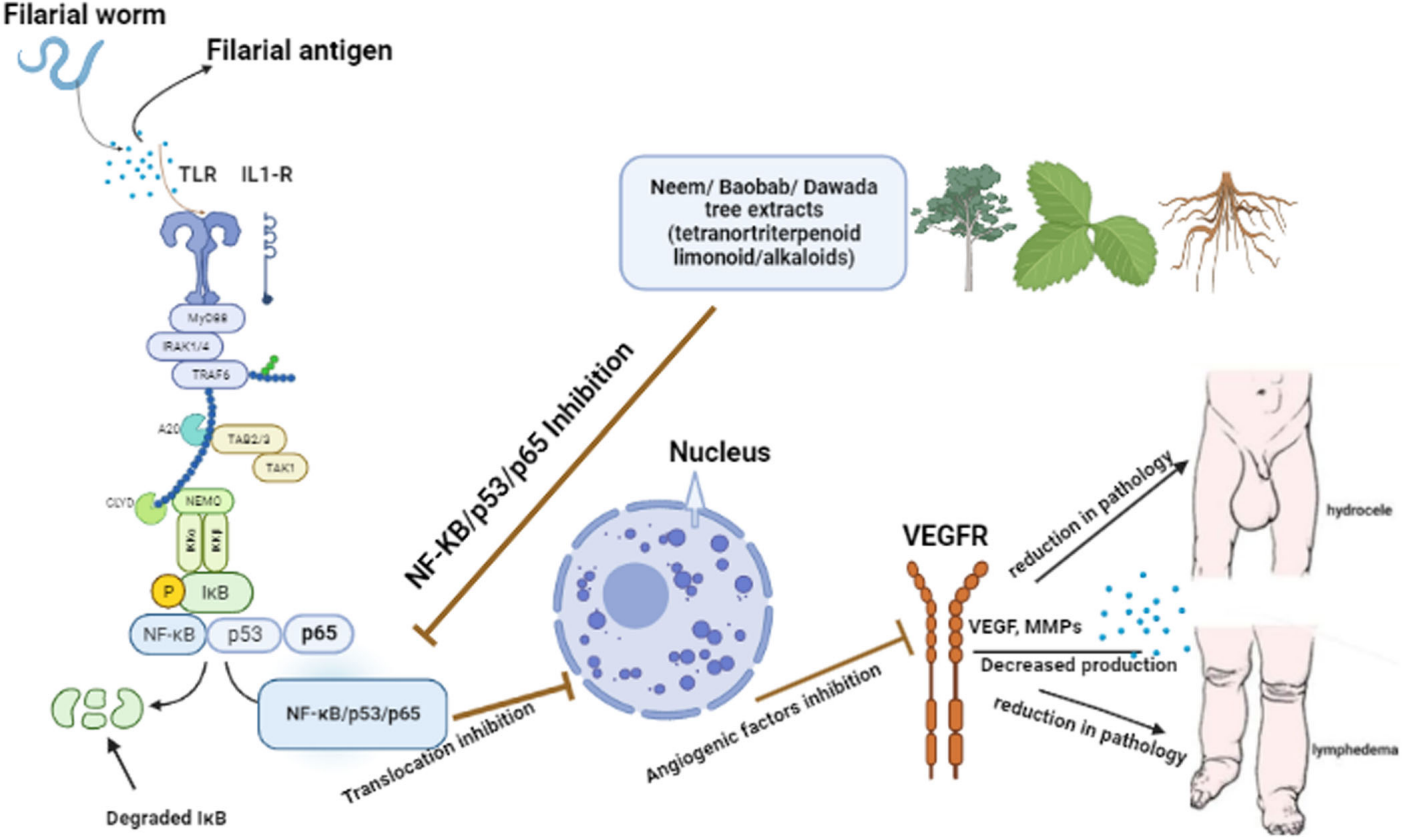
Possible anti‐inflammatory action of medicinal plants against lymphatic filariasis (LF). The pathogenesis of LF is proposed to be due mainly to elevated inflammatory cytokines (IL‐1, TNF‐⍺). Compounds from medicinal plants regulate this pathway by reducing inflammation thereby setting inflammation to levels that are not detrimental to one's health. Created with Biorender.com.

The unavailability of vaccine and drugs for this condition increases the demand for cheap and orthodox methods which are antifilarial in nature.^17^, ^18^ Countries like India has been able to use conventional plant‐based methods in targeting the worms in LF. India has rich tradition of using medicinal plants or their products in treating different disease conditions through Ayurveda, Unani, and Siddha systems of medicine.^8^, ^19^ Several plant medicines have been developed and are being utilized in traditional therapeutics.^17^, ^20^ Natural products of plant origin with insecticidal properties have also been tried in the past for the control of a variety of insect pests and vectors.^2^, ^17^, ^19^, ^21^ Several antifilarial agents containing pentacyclic triterpene and oleanolic acid have also been discovered through research on medicinal plants used by local healers.^17^, ^19^, ^22^ Natural plant products from *Azadirachta indica A. Juss*, *Parkia biglobosa*, *Adansonia* digitata, and *Ocimum sp* are known to have anti‐inflammatory, antibiotic, anticancer, antifungal, and wound healing effects.^23^, ^24^, ^25^, ^26^ These plants are enriched in Africa and known to have bioactive compounds that could be used in treating several conditions. The aim of this perspective is to share and ignite research interest toward the exploration of African medicinal plants as treatment options for individuals with LF and others living with NTDs (Figure 2). Focusing on isolated compounds, medicinal plants and folklore plants having antifilarial activity will help in reducing the burden of providing drugs for LF treatment.

## 2 CONSTITUENTS OF NATURAL PLANT PRODUCTS AGAINST LF

*Azadirachta indica A. Juss* locally known as Neem tree (family meliaceae) is found mainly in the tropical regions, especially in West Africa and India. Animal model studies using *Azadirachta indica A. Juss* have shown that it plays an anti‐inflammatory role by reducing oedema through intramuscular and skin punch biopsy applications using the extracts.^27^, ^28^ Pathway analysis using in an animal model has established that the chief constituents (Azadirachtin, nimbolide, salannin, and quercetin) of the tree play pivotal roles in anticancer management through the modulation of various inflammatory pathways including p53, NF‐*κ*B, and VEGF, but the exact molecular mechanism used by these constituents in the prevention of pathogenesis is not yet fully understood. Other studies have also confirmed its potency against filarial parasites and their vectors.^26^, ^29^ Neem is also known to have antimicrobial properties through its inhibitory effect on microbial growth by showing efficacies against *Staphylococcus aureus* and Methicillin‐resistant *Staphylococcus aureus* (MRSA) with greatest zones of inhibition noted at 100% concentration.^26^ Tannins and flavonoids are the major polyphenols present in the bark of the trunks of *P. biglobosa*. These constituents have shown to be effective in the treatment of inflammatory diseases.^30^

This approach could be explored in the treatment of inflammatory conditions such as is seen in LF pathologies. Various parts of *P. biglobosa* (family Fabaceae, locally called Dawadawa tree in Ghana) have good antimicrobial activities against some bacteria strains.^31^ So far, there is still no clinical study conducted on the plants to investigate activity in most human diseases including LF.^31^ With the inability of the filarial drugs to cure pathologies in LF, a look into this plant's constituents (flavonoids, saponins, tannins, and triterpenes) with its reported higher antimicrobial properties could reveal a higher activity against the *Wolbachia* bacteria which is known to have an endosymbiotic relationship with the filarial worm.

**Figure 3:**
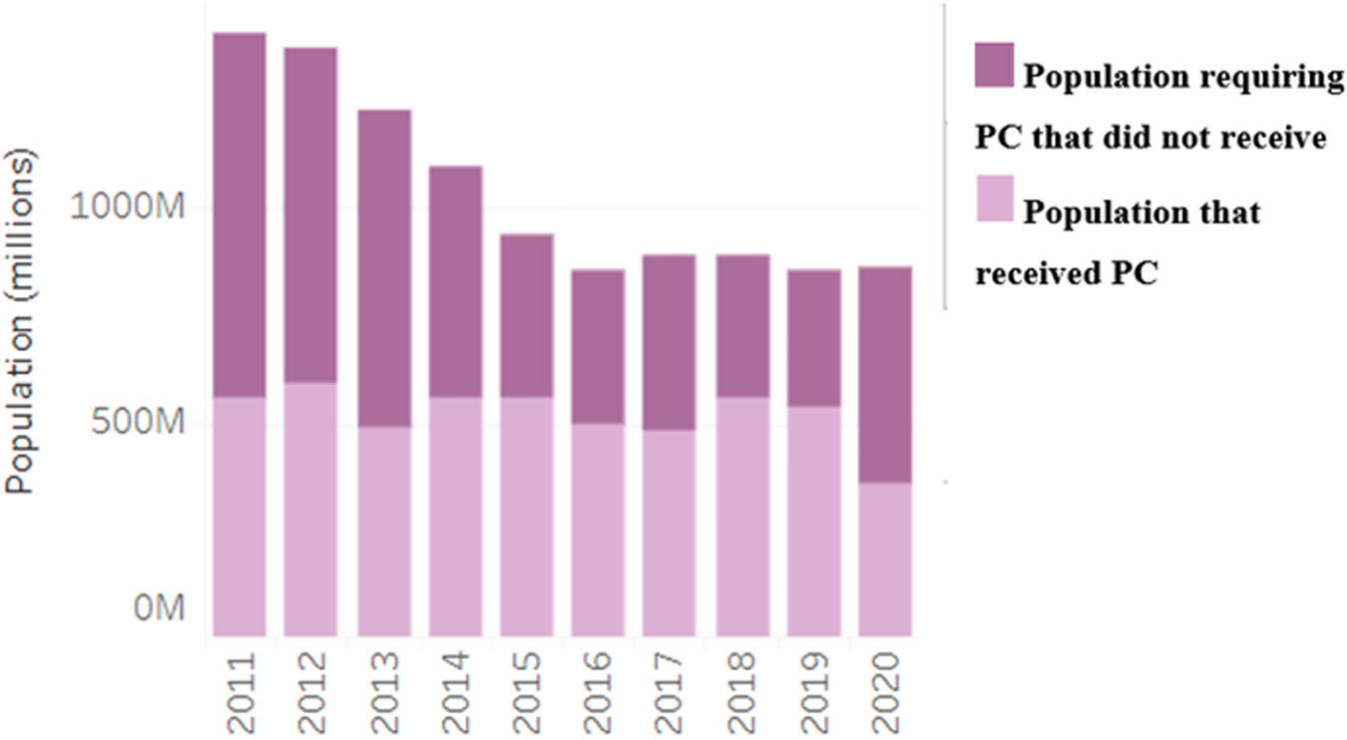
Preventive chemotherapy (PC) coverage per year for lymphatic filariasis.

*Adansonia digitata* (Baobab) known locally as monkey bread tree is a forest tree, primarily in Africa and Asia, in the family Malvaceae. Fruit pulp extract of baobab possesses anti‐inflammatory, hepatoprotective, anticlastogenic, and other medicinal properties.^32^ Baobab affords anti‐inflammatory compounds (campesterol, cholesterol, isofucosterol, β‐sitosterol, stigmasterol, and tocopherol) that can aid in several conditions, from injuries, aches, and pains to stomach upset and respiratory conditions. Polysaccharides from *Adansonia digitata* purified through permeation chromatography have proven to be a potential antioxidant and anti‐inflammatory food supplement.^33^ It is paramount to reduce or regulate inflammation to levels that is tolerable for the body, therefore this plant and its components could help reduce elevated inflammation as seen in individuals with LF pathologies (Figure 3). This inspired a study reporting on the efficacious therapeutic activity of Azadirachtin against LF. It further indicated that a significant tetranortriterpenoid phytocompound found in *Azadirachta indica*, showed efficacies against the filarial parasite *Setaria cervi* in vitro.^34^ Thus, remedies for LF pathology should be directed towards interventions that are known to invoke less or moderate inflammation. Data has shown that not only are individuals with LF pathologies faced with unfathomable societal stigma and discrimination, also they experience acute filarial attacks due to systemic inflammation. Exploration of the baobab tree to ascertain the exact inflammatory pathways targeted by the constituents of the plant could help ameliorate this occurrence which is mostly at its peak in the wet/rainy seasons.^35^

Essential oils from three *Ocimum* spp *from the family* Lamiaceae (*Ocimum gratissimum* [OG], *Ocimum tenuiflorum* [OT,] and *Hyptis suaveolens* [HS]) have been shown to have repellent activities against vectors of malaria and LF.^36^ This plant is locally known in Ghana as “Akoko mesa.” It may be worthwhile to reinvestigate the potential of essential oils derived from other *Ocimum sp* for possible production of mosquito coils, which could substantially be utilized in repelling blood‐seeking mosquitoes and break the vector‐human contacts (Table 1).

**Table 1:** Plants and their constituents targeting various pathways for possible activity against LF.

Plant species	Local name	Activities	Constituents	Pathways	Reference
1. *Azadirachta indica A. Juss*	Neem tree	Anti‐inflammatory/antimicrobial	Azadirachtin, nimbolide, salannin, and quercetin	P53, NF‐κB, 100% zone of inhibition against MRSA	[26, 29]
2. *Parkia biglobosa*	Dawadawa tree	Antimicrobial	Flavonoids, saponins, tannins, and triterpenes	Microbial RNA	[31]
3. *Adansonia digitata*	Monkey tree	Anti‐inflammatory, hepatoprotective, anticlastogenic	Campesterol, cholesterol, isofucosterol, β‐sitosterol, stigmasterol, and tocopherol	Metabolic pathway/apoptotic pathway	[33]
4. *Ocimum sp*	Akoko mesa	Repellent activity	Iron, magnesium silicates, plagioclase pyroxene.	Biocontrol (larviciding)	[53]

## 3 PHARMACOKINETICS OF PLANT MEDICINES AGAINST LF

There is an urgent need to expand the available pharmaceutical repertoire. This must be preceded by experiments on the potencies, activity doses, and toxicity of potential medicinal plants. Studies on the antimicrobial activities of medicinal plants (neem oil extracts, *P. biglobosa*, etc) and phytochemicals using in‐vitro methods (broth dilution, disc or agar diffusion, and agar overlay assays) are used to determine the minimum inhibitory concentration and minimum bactericidal concentration of each treatment.^30^, ^32^, ^37^ Alternatively, in vivo model studies have been implemented to reflect human infections and disease testing; these models include intraperitoneal or intravenous injection, or oral or gastric administration of plant extracts in mice, rats, guinea pigs, and rabbits.^38^

While thousands of people die each year from supposedly “safe” over‐the‐counter remedies, deaths or hospitalizations due to medicinal plants are so rare to find. This notwithstanding, the appropriate volume of medicinal plants whether used as food or supplement is of great concern in both humans and animals. A study reported pathological neurological disorders in horses following a large intake of fresh *Bambusa vulgaris* leaves.^39^ Surprisingly, the aqueous extract of this same plant is a popular antimalarial medicine in Ghana.^40^ Dye exclusion and MTT assay showed the potency of azadirachtin against *S. cervi* with a median lethal dose (LC_50_) of 6.28 μg/mL for microfilariae (mf), and 9.55 μg/mL for adult parasites. A mouse model study on the toxicity of neem trees showed that clinical dosage should be less than 1600 mg/kg/day to prevent organ damage.^41^ Elsewhere, it has been concluded on the toxicity of *P. biglobosa* that 1800 mg/kg and 1600 mg/kg were the intraperitoneal lethal dose 50 (LD_50_) for aqueous extract from roasted and fermented seeds, respectively.^42^ This implies nontoxic dose should be less than the above doses. Moreover, after 60 min of oral administration of ethyl acetate extracts of *Ocimum sp*, mice with edeama experienced less inflammation.^43^ Methanol extracts of *Dipterocarpus zeylanicus (*triterpene saponins) were investigated for macro and microfilaricidal activity through guided chromatography and shown to be potent against *Setaria digitata*.^44^

However, most of these studies have not been replicated in humans to verify their efficacies, especially in LF. This calls for experts' attention on the effect of these plants to help counter the burden of NTDs on the African continents.

## 4 PROSPECTS OF MEDICINAL PLANTS AGAINST CHRONIC WOUNDS AMONG FILARIAL LE PATIENTS

LE manifestation in LF patients is mostly accompanied with the presence of lesions. The delayed healing of these lesions is mainly due to the impaired immunity and increased risk of infections associated with secondary LE, thereby the increased chronicity and difficulty in the management of filarial wounds. The crushed seeds of *P. biglobosa* has proven to have wound‐healing abilities toward snakebit among the Fulani tribes of northern Nigeria.^45^ In LF there are several reports of sustained bacterial infection as cause of chronic wounds in LF pathologies, and this could be mitigated through treatment with *P. biglobosa* which has been shown to have wound healing activity.^46^ Study has also shown that aqueous extracts of neem leaves have wound‐healing ability and this is an appropriate option, preferred for its naturality, ease of access, and is safe, with no known adverse effect.^47^ Further investigations into the wound healing abilities of African medicinal plants as cure for LF wounds would bring relief to the debilitating conditions of LE patients with chronic wounds.

## 5 CURRENT CHALLENGES TO THE USE OF PLANT PRODUCTS IN LF

Although a large population of Africa rely on medicinal plants for primary healthcare, the scientific data on such plants remain limited. The safety profile of these medicinal plants is delineated especially for helminthic infections. Studies have found toxic concentrations of some plant extracts (*Khaya senegalensis, Aframomum melegueta*; IC_50_ of 61.1, 79.7, 61 μg/mL, respectively) used as treatment of helminthiasis. We mention here that although *W. bancrofti* infections account for higher proportion (91%) of total LF infections while *B. malayi* and *B. timori* are responsible for only 9%, data on medicinal plants against LF is highly scarce.^4^ Specifically, research on medicinal plants against NTDs seems to be neglected. Thus, we advocate more works to be done on filaricidal plant extracts on human filarial infections.

## 6 PLANT PRODUCTS AS FUTURE REMEDIES AGAINST FILARIASIS

The availability and search for an effective drug against LF dates to the 1990s. This has been an endless battle with some successes; however, high throughput strategies and research needs to be done as WHO could not achieve its target in 2020 (Figure 3).^48^ With the new target set for 2030, plant products should be explored as treatment options for individuals with LF especially in areas that have less MDA treatment coverage. Moreover, as the current mechanism of action of IVM, ALB, and DEC remain unable to clear the adult worm which sustains repopulation of the human host with microfilariae, this calls for more attention toward plants that have shown tremendous anti‐inflammatory, anticancerous and antimicrobial properties in animal models.^30^, ^33^ Data on the biomedical activity of these medicinal plants against LF in human populations is inadequate, thus research in this area seems neglected making the disease more neglected. With the influx of antimicrobial, anti‐inflammatory, and anticancer activity of some medicinal plants, it has necessitated an inquiry into the effect of these plants and their specific components on LF pathologies.

Another way by which these plants and their extracts can be used in the fight against LF is by using their components for vector control.^4^ One of the strategies set by the WHO in eliminating LF is mosquito control. Mosquito control is a supplemental strategy supported by WHO. It is used to reduce transmission of LF and other mosquito‐borne infections. A study has proven the ability of these extracts as an effective vector control strategy.^4^ The use of these plants' products for vector control could help in protecting our environment and reducing side effects of chemical‐based insecticides. This has also created gaps for exploring environmentally friendly methods in fighting diseases and their vectors. Furthermore, proper control of the filarial vector can be achieved via careful design of extraction and administration processes such as use of efficient bio‐chemical solvent extraction methods, preferably hydrophilic solvent, and logically controlled doses.^4^

Expanding the coverage of food additives with medicinal benefits could help accelerate drug discovery efforts, especially in LF. *P. biglobosa*, which is usually used as local condiments in Africa, especially Ghana, has also been documented to have antimalarial activity during in vivo and in vitro studies.^24^, ^49^ The importance of *P. biglobosa* seed in African traditional medicine and food is undeniably wide and is of prehistoric origin. The growing interest of scientific focus on medicinal plants has contributed to the various experimental evidence including the anti‐inflammatory, antihypertensive, antidiabetes, antioxidant, and antimicrobial activities of these plants. Their activities are mainly linked to their phytochemical composition. Nonetheless, much work still needs to be done regarding the route of administration, toxicity, dosage, regimen, plant parts efficacies, and the medium of extraction.

## 7 PLANT ENDOPHYTIC FUNGI AS A SOURCE OF BIOACTIVE PLANT PRODUCTS AND FUNGAL PATHOGENS AS FACILE MODEL FOR EUKARYOTIC PATHOGENS

The development of low‐cost plant medicine‐based treatment of options ought to include the important endophytic fungi that are harbored by the key plants that serve as the leading treatment options.^50^ An example is found with case of the Yew tree that is the sources of the vital cancer drug taxol. Several endophytic fungi isolated from this tree have been shown to also produce taxol.^51^ While the cultivation of tree plants is subject to long generational time, that of endophytic fungi is short and amenable to the culturing in fermenters which can be controlled to produce desirable quantities of useful compounds.

Aside, endophytic fungal, fungal pathogens such as *Candida albicans* can be employed in large‐scale studies as general model for eukaryotic pathogens. This system offers a simple and low‐cost bioassay platform to test plant medicine preparations and all related samples and isolated compounds. Prescreening with fungal pathogens allows for large libraries of plant materials and compounds to be reduced to a small package of focused and enriched samples to be tested on filarial worms and their eggs.^52^

## 8 CONCLUSION

Out of the 860 million and more people who required treatment for LF in 2020, the WHO progress dashboard indicates that only 360 million people received treatment. This is mainly due to the pressure mounted on WHO to supply these drugs (IVM, ALB, and DEC). This review presents possible insights on the efficacy of medicinal plants as direct filaricidal biomedicine and/or those employed as vector control agents. Methods used for biochemical extraction, screening procedures and structure elucidation of the bioactive compounds to validate the efficacy of plant extracts as another treatment option for LF should be investigated through further animal and human studies.

## AUTHOR CONTRIBUTIONS

**Fatima A. Fordjour**: Conceptualization; resources; validation; visualization; writing—original draft; writing—review and editing. **Priscilla Osei‐Poku**: Writing—original draft. **Afua K. A. Genfi**: Conceptualization; Writing—review and editing. **Kwaw G. Ainooson**: Supervision; visualization; writing—review and editing. **Kingsley Amponsah**: Supervision; writing—review and editing. **Patrick K. Arthur**: Supervision; writing—review and editing. **G. Richard Stephenson**: Supervision; writing—review and editing. **Alexander Kwarteng**: Supervision; writing—review and editing.

## CONFLICT OF INTEREST STATEMENT

The authors declare no conflict of interest.

## TRANSPARENCY STATEMENT

The lead author Fatima Amponsah Fordjour affirms that this manuscript is an honest, accurate, and transparent account of the study being reported; that no important aspects of the study have been omitted; and that any discrepancies from the study as planned (and, if relevant, registered) have been explained.

## Data Availability

The data are accessible via referenced articles. Any further data regarding the article can be made available upon reasonable request to the corresponding author.
